# Quantum ontology without textbooks. Nor overlapping

**DOI:** 10.1007/s13194-024-00573-w

**Published:** 2024-02-21

**Authors:** Cristian Lopez

**Affiliations:** Université de Lausanne, Lausanne, Switzerland

**Keywords:** Quantum ontology, Meta-ontology, Quineanism, Carnapism, Fundamentality, Effective theory, Non-relativistic quantum mechanics

## Abstract

In this paper, I critically assess two recent proposals for an interpretation-independent understanding of non-relativistic quantum mechanics: the overlap strategy (Fraser & Vickers, 2022) and the textbook account (Egg, 2021). My argument has three steps. I first argue that they presume a Quinean-Carnapian meta-ontological framework that yields flat, structureless ontologies. Second, such ontologies are unable to solve the problems that quantum ontologists want to solve. Finally, only structured ontologies are capable of solving the problems that quantum ontologists want to solve. But they require some dose of speculation. In the end, I defend the conservative way to do quantum ontology, which is (and must be) speculative and non-neutral.

## Introduction

There seems to be a symptom of “metaphysical” fatigue in the field of quantum ontology. The feeling is comprehensible: quantum mechanics was formulated roughly one-hundred years ago, and we don’t even know yet whether quantum mechanics is about a single world or many! It is true that we have many good working proposals, but no one has categorically won the quarrel so far. The situation is a bit worse when we realize that there seems to be no empirical or logical way to solve key issues in the field, but that we should rather go deeper and deeper into speculations, balancing various non-empirical criteria for theory choice.

This situation directly threatens scientific realism. Quantum mechanics is one of our most successful scientific theories, but it nonetheless suffers from an acute case of ontological underdetermination. The problem is that we have many different (even contradictory) ways to interpret non-relativistic quantum mechanics^1^ (NRQM henceforth): the so-called ‘interpretations’, ‘alternative quantum theories’, or ‘speculative, ontic interpretations’. It is understandable that scientific realists and quantum ontologists alike feel a bit hesitant about it. Some scientific realists just give up on being realist about NRQM (Hoefer, 2020). Others resort to non-empirical virtues to dissolve underdetermination (Callender, 2020). But the hesitancy and the feeling of metaphysical fatigue have taken a novel, well-defined form lately. The task is to identify interpretation-neutral claims that turn out true in *all* interpretations of quantum mechanics, avoiding speculation and endless quarrels. This would provide a solid ontological basis on which realist commitments can rely safely. The search for a ‘neutral’ quantum mechanics is not new (Cordero, 2001, Belousek, 2005), but it has gained force in the last years because two attractive proposals: the overlap strategy (OS henceforth, see Fraser & Vickers, 2022) and the textbook approach (TQM henceforth, see Egg, 2021). Although OS and TQM differ from each other, I regard them as part of the same “neutral approach to quantum mechanics” that they pursue, and I refer to them as ‘neutral approaches’.^2^

The aim of this paper is to critically evaluate neutral approaches. In particular, I argue along two related, but different lines. First, the neutral approaches assume a meta-ontological framework that can at best deliver flat, structureless ontology. I take this meta-ontology to be inspired by the Quinean-Carnapian meta-ontology that has shaped ontological investigations since the 50 s. I contend that because of this meta-ontological framework, OS and TQM fall short to deliver a quantum *ontology*. In particular, they fall short to even try to solve the most pressing issues in quantum ontology (e.g., the measurement problem). I draw the attention to an implicit confusion between two different projects. One is the project of defending scientific realism in the quantum domain. The other is the project of providing an ontology for quantum mechanics. While I do agree that neutral approaches might provide an answer to the problem of scientific realism, they fail to provide an answer to the problem of a quantum ontology.

My second line of argumentation favors speculation when it comes to quantum ontology. In this aspect, all the old quantum ontologies, such as Everettian quantum mechanics (in its many forms), modal interpretations, pilot-wave theories (in its many forms) and collapse theories, perform much better. But they have done it so because they have abandoned the Quinena-Carnapian meta-ontological framework and pursued structured, richer ontologies. Only by doing so, they are able to elucidate what the quantum world looks like, how NRQM connects with the physical world, and to offer a coherent understanding of the quantum ontology. In the end, I defend speculation because I defend the old ontological ‘conservative track’ that most alternative, speculative quantum theories have walked on so far.

The article is structured as follows. In Section 2, I describe the ‘conservative track’ and the main issues that a quantum ontology attempts to solve. In Section 3, I introduce OS and the TQM. In Section 4, I show that they rely on a Quinean-Carnapian meta-ontology that yields flat, structureless ontologies, where existence questions become central. In Section 5, I argue why I find neutral approaches unconvincing and manifestly weak when it comes to quantum ontology. In Section 6, I defend the necessity of speculation to build *structured* ontologies. In the end, I defend the necessity of speculation and remaining on the conservative track when it comes to quantum ontology.

## Quantum ontology –The “conservative track”

Quantum theories, broadly understood, are probably among the most successful theories we ever have. However, they do not form a compact, unified corpus, but a truly massive set of various theories, models and techniques that account for numerous phenomena and experiments at different scales in the quantum regime. These models and techniques range over relatively simple and low-energy quantum systems (e.g., non-relativistic quantum mechanics) as well as over many-particle systems within the relativistic regime (e.g., quantum field theories). Beside the differences among quantum-mechanics models and theories, there is some agreement that many of the most bewildering features already show up in NRQM, even in its most simple models.

What is the most general problem? Well, the problem is a tension between *pragmatism* and *understanding*. Whereas NRQM and its phenomenology (understood in the experimental sense) work stupendously well to account for empirical data, it does not deliver a clear picture of what the world is like if the theory is taken to be (approximately) true. This is mainly so because it does not have a straightforward way to connect the formalism with the physical world. So, if one believes that a full-fledged physical theory (as I do) should desirably provide not only a powerful formalism to explain phenomena, to generate phenomenological models that fit the data, and to make several predictions, but it should also provide a clear picture of what the world is like if the theory is approximately true, then NRQM is not just a good physical theory –it performs very highly in the pragmatic aspects, but it fails in providing such a picture.

Solutions to this general problem have come from the *alternative* quantum theories or speculative, ontic interpretations (e.g., Bohmian Mechanics, GRW models, Everettian Many Worlds, or Modal Interpretations, among others). The motivations and differences among these theories have been already extensively discussed in the literature, so I won’t get into details here. I just want to remark that all of them are attempts to solve specific conceptual issues, to tell a story about what’s going on in the quantum realm, to connect NRQM formalism with the physical world, and to deliver a picture of what the quantum realm is like. This is just a wordy way to say that they all are attempts to solve the so-called ‘measurement problem’^3^ (see Maudlin, 1995; Wallace, 2008). For it, they in general provide an ontology that explains in a coherent way how the theory connects with the external world and sometimes they modify the dynamics of NRQM. In any case, all the attempts to solve the measurement problem, to the extent that they are different solutions, portray different ontologies.

But how do quantum ontologies look? They are not merely a shopping-list of existence claims, but they try to structure the ontology by distinguishing between what is fundamental and what is not. There are at least two senses of ‘fundamental’ at play that have not been carefully distinguished. Many of the proponents of neutral approaches claim that they are not pursuing a ‘fundamental ontology’: Egg defends an effective, non-fundamental ontology greatly, though not exclusively, based on quantum-mechanics textbooks; Fraser and Vickers seek for interpretation-neutral claims that must not be taken as ontologically fundamental. Their view is well motivated. For instance, Egg argues that NRQM is not a fundamental theory, so why should we suppose that it gives us a fundamental ontology? Even though the problem of what is a fundamental theory is not so straightforward (under strict standards not even quantum field theories can be regarded as fundamental, see Crowther, 2019), I agree that it is not reasonable to expect NRQM to settle what is *the* fundamental ontology.

Yet, it does not mean that the concept of ‘fundamental’ must be just rejected. It does play a role in quantum ontologies, even though they do not settle what is *the* fundamental ontology. So, it is useful to distinguish between what I call ‘fundamental *simpliciter*’ and ‘*intra*-theory or *intra*-ontology fundamental’ (‘intra-fundamental’ for simplicity).^4^ Tuomas Tahko proposes a sense of ‘fundamental’ as a “common minimal basis” (CMB), that is, the set of entities, relations, and properties that determines everything else (see Schaffer, 2003, Tahko, 2014). Beyond technicalities, what is fundamental should be taken in terms of ‘building blocks’ that determine the rest of the ontological building and as *complete* descriptions of the rest of the ontology. When I talk about ‘fundamental simpliciter’, I refer to *the* CMB of reality as a whole, as *the* complete description of reality. But when I talk about ‘intra-fundamental’ I refer to a CMB relative to a theory and as a complete description of the quantum ontology. So, while it may be true that quantum ontologies are not *the* fundamental ontology in the sense that they do not provide the CMB of reality, they are indeed structured in terms of an intra-fundamental and an intra-non-fundamental ontology. This will be clearer in Section 5, but this distinction should be kept in mind.

It is important to note that alternative quantum theories mostly disagree on what such a CMB and its dynamics are. They hardly disagree on the rest of ontological claims, such as whether an electron has gone through a Stern-Gerlach, as what we observe, etc. To put it differently, the problem arises because there are different ways to specify which is the intra-fundamental ontology of quantum mechanics, while keeping the rest of the ontology fixed to a good extent.

## The overlapping strategy and the textbook account

Different quantum theories deliver different ontologies. Many have seen that this is a problem of ontological underdetermination. Then, it seems that the problem is no longer how to make sense of NRQM, but how *to choose* between alternative ways to make sense of NRQM. It is, ultimately, a problem of theory choice on the base of a severe case of underdetermination by evidence.^5^

Neutral approaches diagnose that the problem of underdetermination has stimulated speculation. But underdetermination and speculation have fed anti-realist concerns on the quantum domain (see Hoefer, 2020). In this vein, neutral approaches are ways to save scientific realism from antirealism fuelled by the problem of underdetermination. Yet, they do have some implications that transcends the scientific realism debate. At different degrees, they commit themselves to provide a quantum ontology and also recommend some overarching meta-ontological principles for quantum ontology. As I said before, OS and TQM are the most recent, and probably the best, exponents of neutral approaches. So I focus on them.

### The overlap strategy

The OS has been mainly defended by James Fraser and Peter Vickers in the 2022 paper “Knowledge of the quantum domain: an overlap strategy”. As a solution to the problem of underdetermination, the OS basically consists in identifying some descriptive, interpretation-neutral statements that all speculative, ontic interpretations (as they call them) agree. This agreement is not merely empirical, nor based on trivial, empty claims, but substantive enough to deliver genuine knowledge of the quantum domain. This, the authors argue, lays out a basis on which scientific realism can be suitably defended, avoiding the speculations and the epistemic risk of alternative quantum theories:

We consider plumping for a particular ontic interpretation, on whatever grounds, to be a poor option for someone wishing to defend knowledge claims about the quantum domain. Doing so is simply too epistemically risky given the theoretical and empirical information that is currently available (Fraser & Vickers, 2022: 8).

Their assumption is that the scientific realists do not want to run such an epistemic risk, but she wants to make “safe commitments that can be trusted to stand the test of time” (ibidem).

Which is concretely the strategy? It starts off by taking some very basic statements upon which all quantum theories agree (for instance, about energy state transitions of Hydrogen). Then, the terms that appear in the description are broadened in such a way that “straddle” all ontic interpretations, without rendering them trivial (see Fraser & Vickers, 2022:13 for an example). For instance, if in the description occurs the term ‘electron’, it is broadened in such a way that it becomes less specific and more general but escapes the problem of underdetermination as it omits details upon which alternative ontic interpretations could disagree. For this linguistic maneuver to work out, they rely on (i) taking the neutral descriptions as ontologically non-fundamental, (ii) adopting common practice in daily life and philosophy of language (in particular, how causal and descriptivist theories fix the reference of terms), and (iii) drawing a distinction between ontology and semantics, or truth-makers and truth-conditions—the overlap strategist can attribute truth value to propositions without delving into their fundamental truth-makers.

In the end, the OS advises taking a case-by-case approach. It means to identify in each alternative quantum theory and in their best-supported models for a quantum phenomenon those claims about the unobservable that are true in all of them. This will allegedly give us a list of interpretation-neutral statements that deliver substantial knowledge of the quantum ontology but avoid the problem of underdetermination. They recognize that the ontological status of the wave-function (or quantum state, more generally) and the details of the dynamics might mar the program. But the strategy, once again, is to remain neutral on those conflicting propositions, searching for the agreement. This is brought about by formal similarities of the quantum state across alternative quantum theories, decoherence, ‘effective’ collapses, and a general, interpretation-neutral view of quantum possibilities. Summing up, they say:

“Our suggestion is that we can understand statements about the wave-function as encoding claims about the physically possible states and evolutions of a quantum system while remaining open to different, more precise, analyses of the nature of these ‘possibilities’ provided by particular ontic interpretations” (Fraser & Vickers, 2022: 21).

In this way, a solid basis of knowledge claims can be guaranteed to the scientific realist and a quantum ontology deployed. It can be achieved by a certain appropriate level of abstraction, that is not dramatic, nor renders trivial knowledge.

### The textbook approach

In a 2021 paper called “Quantum Ontology without Speculation”, Matthias Egg defends what Craig Callender has called “the textbook approach” (Callender, 2020),^6^ TQM. Egg’s defense of the view not only proposes a non-speculative substantive quantum ontology, but also flags a normative standard for quantum ontology in general. Less speculation would allow us to focus on neutral statements about quantum ontology, upon which even the competing speculative, alternative theories should agree. This explains why Egg’s view has been considered as a “non-compulsory extension of the overlap strategy” (Fraser & Vickers, 2022: 11). But it goes a step further as it is not meant to merely deliver some relief for the scientific realist when it comes to quantum mechanics, but to offer a *substantive* quantum ontology that puts speculative approaches to rest.

Egg’s view defends a methodological thesis and an ontological constraint. As for the former, it says that ontology should foremost be informed by our best current physical theories. The qualitative predicate ‘best’ must be couched in terms of “the most empirically successful”. TQM claims that we should regard NRQM as it is introduced in quantum–mechanical textbooks (in its “somewhat messy and recipe-like form”, Egg, 2021: 6) as responsible for the empirical success of quantum mechanics. Therefore, a non-speculative quantum ontology is to be, to a good extent, based on TQM since the rest of the quantum theories posit further ontological content that does not boost NRQM’s predictive capacity and empirical adequacy (this idea is clearly in line with Saatsi’s progress realism, see Saatsi, 2020, 2016). In this way, TQM then seeks to safely “fence in” a set of undisputable and neutral statements about quantum ontology. To overstep the fenced-in zone is to step into the realm of endless speculative quarrels, which we should avoid. From this, a methodological thesis can be extracted.

The methodological thesis delivers a series of ontological commitments that are the basis for a *substantive* quantum ontology. Egg recognizes that, at first glance, textbooks are conceptually confusing about many ontological issues, but this is in some sense only apparent. He argues that many of the conundrums in quantum ontology stem from pursuing what he calls a ‘fundamental ontology’. What he means by ‘fundamental ontology’ is what I meant in Section 2 by ‘fundamental simpliciter’. Quantum-mechanics textbooks are certainly unable to provide any fundamental ontology simpliciter, but the blame should not be put on the textbooks, but on the nature of the inquiry. If we want to obtain a substantive quantum ontology upon which most of the alternative quantum theories will agree, we need to abandon the project of providing a fundamental ontology. In order to do that, TQM endorses an ‘effective’ (or ‘functional’) ontology that is non-fundamental (simpliciter).

The idea makes sense because NRQM is *not* a fundamental theory, but an effective one.^7^ An effective ontology, then, focuses on the vastly enough number of quantum–mechanical models, frequently taught in quantum–mechanical textbooks, and see which posits of the theory are doing the predictive and explanatory work. This brings up the ontological constraint I mentioned before: the quantum ontology should circumscribe itself to the non-fundamental posits that are “doing the work” in the predictive and explanatory success of the theory. This leaves out any question about the nature of such posits, or what they fundamentally are.

### Neutral approaches and meta-ontology

Even though neutral approaches are primarily attempts to rescue scientific realism from the problem of underdetermination, they explicitly or implicitly imply a quantum *ontology*. In so far as they do that, they also recommend a meta-ontological framework.

Most of TQM’s meta-ontology is condensed in the following quotes:

Ontology should be informed by our best current theories and that what makes QM one of our best (i.e., empirically most successful) theories is not any of its ontologically kosher (speculative) formulations, but the somewhat messy and recipe-like form in which it appears in textbooks (Egg, 2021: 6)

State vectors (or wave functions) codify the behavior that quantum systems display in virtue of their quantum states in given experimental situations. This is the sense in which the ontology of quantum states is given by what they *do*, namely to bring about specific kinds of behavior in the quantum systems that are in those states (Egg, 2021: 8)

[the effective realist] rejects the fundamentalist’s requirements on a ‘clear interpretation’: as long as TQM precisely informs us about how quantum systems behave as a function of their spin state […] It yields all the ontological precision one can expect from an effective theory like QM (Egg, 2021: 23)

There are three meta-ontological elements that guide Egg’s quantum “textbook” ontology:

- Ontological Naturalism
- Functionalism
- Non-Fundamentalism

According to the fist, ontological commitments should be guided by empirically successful scientific theories, drawing a continuation between the sciences and ontology. According to the second, the theoretical posits that deserve ontological commitments are those that “do the work” in the best scientific theories, in terms of delivering explanatory and predictive capacity. This presumably articulates with the naturalist credence—it is epistemically less risky to commit to those posits that are responsible for the empirical success of the physical theory. Finally, such posits should not be taken as ontologically fundamental simpliciter, but as non-fundamental as was previously mentioned.

The OS as defended by Fraser and Vickers support most of these points but emphasizing different aspects. For instance, they take Egg’s proposal as a suitable meta-ontological framework to resist commitments to a fundamental ontology:

Egg argues that we need to adopt a meta-ontological framework that recognizes a plethora of non-fundamental scientific entities in addition to the fundamental ontology posited by a theory, and that doing so helps us to articulate substantive ontological overlap between the rival interpretations of quantum mechanics (Fraser & Vickers, 2022: 22)

This clearly follows Egg’s support of an effective (or functionalist), non-fundamental ontology, and Fraser and Vickers see it as a viable meta-ontological framework. In addition, they stress that our ontological commitments should be guided by some resistance to take seriously subjective assessment of the extra-empirical virtues. Conflicting evaluations of the value of extra-empirical virtues bring endless disagreements that undermine scientific realism. As they say, the scientific realist “wants to make safe commitments that can be trusted to stand the test of time”, which can only be provided by quarantining the disagreements, and by basing our ontological commitments on the overlaps.

The idea of a non-fundamental quantum ontology is also defended on the basis of resisting ‘semantic reductionism’ (Fraser & Vickers, 2022: 23), which leads to an in-deep metaphysical unpacking of the intervening terms in relevant propositions to their ultimate truth-makers. They believe that meaningful content and truth values can be assigned to such terms and propositions without engaging in such an in-deep metaphysical unpacking. This is a semantic argument against the necessity of a fundamental ontology. As I read it, it is also an argument in favor of the coherence (and maybe the convenience) of developing a non-fundamental ontology for quantum mechanics.

In a 2022 paper, Juha Saatsi has also drawn attention to the meta-ontological framework of neutral approaches, in particular of TQM. According to Saatsi, the way in which TQM conceives of ontological commitments leads to “overly promiscuity”. The problem is that the effective realist’s undiscriminating and inclusive meta-ontology leads to an overpopulation of entities that is in tension with “scientists’ own reasoning about ‘what is real’, which is arguably also capable of accounting for the usefulness of theoretical posits that are not taken ontologically seriously” (Saatsi, 2022: 6). In a few words, Saatsi’s objection is that the textbook account’s meta-ontology is too liberal, forcing us to adopt ontological commitments (although in the non-fundamental simpliciter sense), that most scientists will not be willing to adopt.

## Ontology and meta-ontology

The discussion on meta-ontology is very welcome. In this section, I would like to zoom out the debate to more general meta-ontological considerations in philosophy to better identify some of the general assumptions of neutral approaches. Although ontological questions have been central in philosophy of quantum mechanics, little has been said about general meta-ontological frameworks for quantum ontologies. I think the point is of relevance to assess the place that neutral approaches to quantum ontology occupy in relation to the ‘conservative track’. The relevance lies in the fact that a meta-ontology prescribes which is the goal of ontological investigation as well as the sort of product is expected to obtain. A divergence over meta-ontology is a divergence over what is the *target* of ontological inquiry. In what follows, I argue that neutral approaches implicitly promote a meta-ontological program strongly connected with a Quinean-Carnapian meta-ontological framework. I don’t mean that OS and TQM explicitly adopt such a meta-ontology, but that they adopt many elements that are distinctive of it. In the next section, I argue that the problem is not only that such a meta-ontology falls short in giving a substantive ontology for the quantum world, but it also misses the point of the most crucial issues in quantum ontology.

### The metaphysics mainstream –Quineanism and Carnapism

The Quinean-Carnapian meta-ontological framework (Carnap, 1956; Quine, 1960, 1963) was a reaction against the traditional metaphysics based on conceptual analysis that reigned before the 1950s. This *naturalist* turn in metaphysics was not only a rejection of traditional analytical metaphysics, but also a call for a closer relation between metaphysics and empirical sciences. And although the Quinean and the Carnapian framework were largely taken as rivals, its rivalry circumscribes to specific theses that do not affect a wide core of general agreements. To begin, both views share a pragmatist, anti-traditionalist view of metaphysics. Second, they agree that metaphysical questions are primordially *existence* questions. In the case of Quineanism, “what is there?” (Quine, 1963: I) is par excellence *the* metaphysical question. The means to know it are similar: choose the best scientific theory (in general, your best *physical* theory), cast it into the canonical logic (*first-order* logic), and determine the domain of quantification (the extension of the existential quantifier over bounded variables) that makes the logical reformulation true. The main differences between both approaches are two. One of the differences is whether existential quantification is univocal and ranges over a unified, single domain (Quine) or it ranges over a multiplicity of domains, leading to linguistic-framework multiplicity and ontological pluralism. (Eklund, 2009; Price, 1997). Another difference is that, while Quinean existence questions can be external, Carnapian existence questions can be only internal (if meaningful). That is, in Carnap our commitments with the existence of entities depend on the linguistic framework in which a theory is formulated to talk about such entities. To put it another way, whether *x* exists independently from a linguistic framework is a meaningful question in Quine, but it is not in Carnap.

It is worth dwelling on one of the central agreements: ontological questions are *existence* questions. In Quine, they ultimately depend on empirical investigation (on our best and most successful scientific theories). In Carnap, they ultimately depend on the choice of the linguistic framework, which is mainly driven by pragmatic reasons. Be that as it may, one of the consequences of exclusively focusing on existence questions is that a flat, structureless ontologies is delivered (see Schaffer 2009). Entities, properties, and relations posited either by our best physical theory or by the linguistic framework under consideration are simply listed as part of our ontological commitments. They could be endorsed externally (as in Quine), or internally (as in Carnap). Either in the form of one flat ontology or multiple flat ontologies, metaphysical questions do not concern any *structure* among the items of our ontology, nor *dependence* relation among existents. To do ontology is to list ontological commitments -–everything exists equally, without qualification, in a sort of “ontological democracy”. The reason is easy to see: Both views simply lack the means to obtain a *structured* ontology^8^; any order, relation or hierarchy among existents will overstep the naturalist preaching since it would entail distinguishing layers, relations between layers, and so on. This will be important for Section 5 and 6.

### Neutral approaches –*a mélange* of Quineanism and Carnapism

Neutral approaches encourage us to search for a neutral basis of agreement to adopt ontological commitments. This resembles the neo-Carnapian “easy approach to ontology”, which starts off from neutral, uncontroversial claims (Schiffer, 2003; Thomasson, 2009). But this also entails distinguishing empirically grounded commitments from ungrounded speculations, which resembles the central tenets of Quineanism and Carnapism as sketched above. Both OS and TQM endorse the idea that ontological commitments should be informed by our best current theories, which clearly fits in the Quinean naturalist tradition. Notably, what makes a physical theory “the best” is to be fleshed out exclusively in terms of empirical success. This could take the form of adopting ontological commitments to the theoretical posits that “actually perform the explanatory and predictive work” (Egg, 2021: 22), or of taking a case-by-case approach “looking at the best-supported models of a given quantum phenomenon” (Fraser & Vickers, 2022) to search for a neutral basis of agreement. Even though I agree that these recipes might lead to an overinflated ontology, it does refrain from adopting ontological commitments to whatever does not boost the empirical content of the theory. In Egg’s view, such ontological, extra-empirical content leads to ontological speculation, and consequently, unsolvable debates.

Focusing on meta-ontological aspects, neutral approaches look in general like the following. NRQM is our best empirically adequate theory about a large set of phenomena. There are undisputable statements that are putatively true, involving terms referring to wave-packets, electrons, energy, transitions, collapses, experimental arrangements involving measurements of spin in different directions, polarization effects, and so on. In order for us to extract ontological commitments, it is only required to do two things:

(i) Deflate (or broaden) the meaning of the terms, so that they can still be meaningful and true. In doing so, it is possible to find an empirically meaningful basis of interpretation-neutral knowledge claims. Note that the TQM takes the same strategy, but it points to quantum-mechanics textbooks as the place where putative overlap of rival quantum theories can be found.
(ii) Take such statements and broadened terms and investigate the implicit existential quantifiers. For instance, if such propositions involve empirically adequate statements about wave-packets, then they exist because we are existentially quantifying over the terms. Therefore, neutral approaches are ontologically committed to wave-packets. Of course, questions about whether the wave-function is complete, whether it represents a field in a high-dimensional space, etc., are beyond neutral approaches’ scope, since they involve speculation and doing quantum metaphysics. In any case, the methodology *to extract* ontological commitments is essentially Quinean in spirit.^9^

Naturalism here goes hand-in-hand with anti-speculation. This is specially emphasized in TQM–what draws the line between a *non-speculative* and a *speculative* ontology is empirical success. If a proposition *p* does not augment the empirical content of the theory, then it is speculative, and thereby, to be discarded. This is also in keeping with Quine’s methodological continuity between science and philosophy. Conforming with TQM, quantum-mechanics textbooks balances optimally well such a set of propositions: it includes the optimum number of statements to guarantee empirical success and to avoid speculation (i.e., they do not introduce ontological claims that do not expand the empirical basis of the theory). OS disagrees with this point as its defenders do not believe that quantum-mechanics textbooks are the right place to look for overlapping. They rather recommend a case-by-case approach that ranges over all the ontic interpretations. This has a Carnapian flavor. First, a substantive ontological proposition should boost the empirical content; otherwise, it is at best a metaphysical cumbersome (re)formulation of the theory. Secondly, any ontological question is to be answered within the linguistic framework of non-relativistic quantum mechanics, which was already assumed to be the best physical theory to explain and predict some phenomena.

Most importantly, the neutral approaches adopt the central meta-ontological thesis of Quinean and Carnapian meta-ontologies: the inquiry in quantum ontology only concerns *existence* questions, either in the internal or external sense. Ontologists’ task is thus to make lists of the existent things (or properties) that NRQM involved in its best formulation. In the defense of a “neutral” quantum ontology, neutral approaches give us a recipe to adopt minimal ontological commitments about what there is that do not generate disputes about competing, speculative quantum theories. There is, however, a price to be paid for neutrality –any neutral quantum ontology is to be flat, structureless. No layers among existents *within* the ontology can be distinguished, nor dependence relations among them can be established.

It can be argued that neutral approaches do not fail to provide us with ontological dependence relations among quantum objects since they provide us with *functional* dependence relations. However, I do not think that is the case. In Egg’s TQM, a functional ontology is an alternative to a *fundamentalist* ontology. That is, a functionalist quantum ontology is a non-fundamental ontology *simpliciter*, in my vocabulary. I fully agree on this, and I think he is right. But this is independent from providing a sense of *intra*-fundamental, which is my requirement in order to obtain a quantum *ontology* properly. In other words, to claim that an ontology is a functional ontology (i.e., a non-fundamental ontology) does not solve the problem to show how *within* the functional ontology a structure can be given (see Section 5 below). Moving from ‘being’ to ‘doing’ does not tell us how to distinguish between different ‘doings’ within the ontology. If such a distinction is not made, then the (functional) ontology is flat, structureless.

It can be counter argued that functionalism can be promoted as an ontological dependence relation within a functional ontology. Nor do Frasser and Vickers, or Egg seem to suggest something like this explicitly, but it is a plausible view. Egg does say that the “location” (in ontological terms) of some functional objects can be done by identifying their realizers. He mentions Esfeld and Deckert, (2018), where properties are conservatively reduced to (localized in) “the configuration of matter points and their change” (Esfeld & Deckert, 2018: 447). It is clear that Esfeld and Deckert pursue a fundamental ontology, and Egg finds the concept of ontology circumscribed to this kind of projects “quite unfortunate”, but it could be held that by adopting functional dependence relations *within* the ontology, a structured ontology is delivered. This might work, but I am a bit hesitant to accept that it works preserving the spirit of neutral approaches. To begin, how are these intra-ontological functional relations established? It can be said that they could come from theoretical and semantic functional relations in the theory. That is, dependence functional relations in the ontology are the reification of functional theoretical and semantic relations in the theory. This is a possibility, but it requires additional argumentation, which will surely incur in some speculation. It is not clear that, for instance, theoretical functional relations can be easily translated into ontological functional relations. Even if this can be done avoiding speculation, it is not clear why alternative formulations of the same theory (which will plausibly yield different theoretical functional relations) would preserve the same ontological functional relations.

Let me be more concrete. For instance, in NRQM as introduced in some textbooks (see, for instance, Ballentine, 1998, Ch. 3.3, 3.4) basic magnitudes for closed, constant-energy system free from external fields are associated with the commutation relations between the generators of the Galilean group (for instance, the three momentum components, the three angular momentum components, the three boost components, and the energy, etc.). The rest of physical magnitudes will be defined in terms of the basic ones (for instance, the three spin components, the three orbital angular momentum, among others). At the same time, there will be operators that commute with all the generators of the group (the Casimir operators of a group, for NRQM the internal energy, the square of total spin, and the mass). It is clear that there is some theoretical structure given by theoretical dependence relations.^10^ But they do not translate without further argumentation into ontological dependence relations. To begin, an argument is required to promote *those* theoretical relations (and not others) as capturing ontological relations. Second, it must be argued why symmetry considerations are good guides for ontology. Third, it must be argued that such ontological dependence relations are *functional* relations within the quantum ontology (and not of a different kind, as grounding relations). To be clear, there certainly is an interesting project here that might deliver a sound and robust ontology. But it lies beyond both TQM’s and OS’s spirit.

I think that there is some overlap between two different projects, not so clearly demarcated: the project of an ontology for quantum mechanics and the problem of scientific realism. Both projects are different and at this point they depart from each other. Neutral approaches, first and foremost, want to escape from the problem of underdetermination, which undermines scientific realism in the quantum domain. In order to do so, they adopt a series of meta-ontological criteria that might fortify the realist attitude by relying on overarching agreements among different quantum theories, focusing on interpretation-neutral knowledge claims. It might be a good strategy to settle the problem of scientific realism in the quantum domain. But, if I am right and they largely rely on a Quinean-Carnapian battery of recipes to extract ontological commitments from NRQM and speculative, ontic interpretations, neutral approaches fall short to provide any substantive solution to the problem of the ontology of quantum mechanics. The key problem to me is that the purpose of quantum ontologies is to address crucial problems that demand *structured* ontologies, for which the Quinean-Carnapian meta-ontology is too weak. To put it differently, neutral approaches deliver a neutral quantum ontology that is too shallow to deal with the problems that quantum ontologists want to address. In this sense, OS and TQM do not even provide competing quantum ontologies. Therefore, the conservative track, regardless how speculative it might be, is to be preferred.

## Ontological clarity and the necessity of speculation

My view is that neutral approaches are good enough if exclusively circumscribed to the scientific realism debate. If the question is whether we should be realist about NRQM, they give a minimal sense in which the answer must be ‘yes’ (although I have some caveats as I show shortly). But if they intend to deliver neutral quantum *ontologies* as alternatives to the conservative track, then the price to be paid is too high. The main problem is that a neutral quantum ontology is unable to provide a convincing, non-speculative answer to the measurement problem (if it provides an answer at all). Nor can it tell a story of the place that the theoretical posits occupy in the quantum ontology and how they relate to macroscopic matter. In short, they fail to connect NRQM formalism with the physical world. If this is so, I submit, they are then much weaker, and more unclear, than speculative, ontic alternatives. Therefore, the price to pay for neutrality is a view too weak to yield a quantum ontology properly. My argumentation is twofold. First, I number a series of issues and ambiguities I find in neutral approaches as they stand. This is done in this section. Next, I show that a different meta-ontology is required to address such issues and to offset the ambiguities. This is done in Section 6.

### Fundamental simpliciter and intra-fundamental

It seems to me that one of the biggest confusions in the neutral approaches is that they fail to draw the distinction that I made in Section 2 between what I have called *the* CMB of reality as a whole (i.e., fundamental simpliciter) and *a* CMB relative to a theory and as a complete description of a quantum ontology (i.e., intra-fundamental). So, while it may be true that NRQM is not the best candidate for a fundamental ontology simpliciter, it does not mean that the distinction between what is fundamental and what is not cannot be drawn within a quantum ontology. Indeed, most successful quantum ontologies depend on drawing such a distinction: some entities, properties, or relations are deemed as irreducible (forming a CMB), while others are deemed as derivatives (or non-fundamental) as they hold some metaphysical relation with the CMB. It is important to stress that to be intra-fundamental does not imply that the theory must be fundamental simpliciter; nor that the CMB of a quantum ontology must also be the CMB simpliciter.

In rejecting neutral ontologies to be fundamental simpliciter, neutral approaches fail to see how important is to nonetheless preserve the idea of intra-fundamental when proposing an ontology for NRQM. Indeed, most quantum ontologies frame themselves within naturalism in the sense of adopting empirical criteria to revise ontological commitments in the light of scientific progress and discovery. Also, most regard NRQM as an effective theory, so that its ontology is bound to be also regarded as non-fundamental simpliciter (in fact, most quantum ontologists do not take NRQM too ontologically seriously and strive for offering relativistic versions of their proposals). But even in a more pedestrian sense, all empirically adequate quantum theories (from one-particle non-relativistic quantum mechanics to the Standard Model) are stricto sensu effective theories (see Crowther, 2019); and thereby, they are non-fundamental simpliciter, too. Therefore, the fundamental ontology simpliciter is then hardly tenable within a strict naturalistic framework. But this does not mean that the label ‘fundamental’ should be eliminated. This would trivialize much of the philosophical exploration in quantum foundations. The clear alternative is to restrict ‘fundamental’ to ‘intra-fundamental’: *a* CMB *within* a quantum ontology.

What I have called ‘the conservative track’ does just that. They hypothesize CMBs that distinguish what is ontologically intra-fundamental from what is intra-derivative *within* a quantum ontology. This is not exclusive of NRQM, but it is also common currency in the ontology of other physical theories. The reasons, it seems to me, lies in the fact that many of the crucial issues in quantum ontology debates (but also in physical ontologies in general) depend on providing *structured* ontologies, in which primitive entities, properties and relations (a CMB) can be identified and related to secondary entities, properties and relations. Such a structured ontology clearly doesn’t imply any commitment to taking the fundamental layer *within* a structured ontology as what is fundamental simpliciter. Otherwise, the quantum ontologies would hardly be empirically revisable. Nor is it incompatible with the fact that the theories at stake are effective theories–it is possible to talk about intra-fundamental in the framework of effective theories (for instance, it is possible to talk about what is fundamental in classical electromagnetism, see Maudlin, 2018). Conforming to this, then, a *structured* ontology can preserve the idea of fundamental, but restrict it to *intra*-fundamental.

My worry is that neutral approaches, in failing to distinguish between these two senses of ‘fundamental’, reject intra-fundamental with the (probably unintended) consequence of hindering the development of structured ontologies. The purpose of introducing structured ontologies is to comprehend how different entities, properties and relations within an ontology glue together, which place they occupy within the ontological landscape. In the previous section I argued how these relations can be introduced at a theoretical level, but their translation into the ontology is far from clear. But there are other problems too. For instance, NRQM deploys a number of Hermitian operators that intendedly represents measurable quantities. Quantum ontologists may wonder: is there any ontological difference among them? The question is not mere philosophical curiosity, but it may play a role in, for instance, solving the measurement problem, that is, in specifying how the NRQM formalism connects with the external world. Bohmian Mechanics, for instance, describes the state of quantum systems in terms of the quantum state (the wave-function) plus the positions of Bohmian particles. Hence, the Bohmian ontology demands to assess the ontological status of all the putative physical properties represented by Hermitian operators. This basically means to locate all the putative physical properties in the ontology, that is, to say whether they belong to the Bohmian CMB, whether they are derivative, or whether they are just unreal (see Lazarovici et al., 2018). But this means to *structure* the Bohmian ontology and not just to provide a flat, shopping-list-like ontology.

Structured ontologies are not about discovering the fabric of reality, but about ontological clarity. Neutral approaches believe that it leads to endless quarrels and speculation. And they are quite right. Take the effective, functionalist ontology of TQM. I said that effective theories (as NRQM) reasonably entail effective ontologies (as that of Egg’s). But I also said that it doesn’t mean that a CMB cannot be distinguished, in the sense of being intra-fundamental. This would make the effective ontology a *structured* ontology, as the argument explored above that promotes theoretical functional relations to functional ontological dependence relations. To emphasize here the point: if this is the case, the effective, structured ontology would be able to distinguish between what is intra-fundamental, what is not, and how they relate to each other. Yet, how is that supposed to be done if we cannot trespass the limits of quantum–mechanical textbooks? Or more generally, how is that supposed to be done without breaking the neutral predicament? Interpretation-neutral knowledge claims (regardless of whether they are to be found in quantum-mechanics textbooks) are too weak to do the work, that is, to provide the means to select the entities, properties, and relations that will form a CMB. But the sort of decisions involved to even separate a system from its environment, or to distinguish ontologically among observables, oversteps what quantum-mechanics textbooks can say and breaks the sought-for neutrality.

As I see it, neutral approaches are caught in a dilemma, *if* they aim to provide a quantum ontology (or at least to lie the basis for one):

(i) If they stop speculating and search for neutrality, then they can at best deliver a flat, structureless ontology. I claim that such a proposal performs very poorly in comparison with the more speculative, ontic alternative.
(ii) But if they want to provide a more substantive, structured ontology, then they need to incur in some speculation, overstepping the limits of quantum-mechanics textbooks, or breaking the promise of neutrality.

It is blatant that the latter amounts to giving up on neutral approaches, but what is wrong with flat, structureless ontologies? This will become clearer shortly and in Section 6 but let me point out the following for the moment. If one of the aims of a physical ontology is to provide a detailed picture of the natural world, then a flat, structureless ontology can hardly achieve that. The main reason for why it cannot do that is because it lacks the resources to identify ontological dependence relations and to solve pressing problems in NRQM. Why is it so important? First, because ontological dependence relations (even in the intra-fundamental sense) explain how different posits relate to each other within the ontology, and that delivers greater ontological comprehension of the quantum ontology. Second, if we do not solve such pressing problems, we then fall prey to the measurement problem and we do not even know how the quantum domain could even make contact with the macroscopic world. Neutrality and non-speculation give us safety ontological commitments, but the price to pay is the absence of a proper ontology. Speculative, ontic interpretations deliver clear ontologies and greater understanding of what the quantum domain looks like and how their posits relate to each other. The price to pay is a structured ontology and with it some dose of speculation. As Fraser and Vickers says, for some this might be too high a price to pay, for me a necessary concession.

### The measurement problem

The measurement problem is the most pressing problem for NRQM. The reason is very simple: NRQM (without any ado) is simply wrong because it predicts states that have never been observed (Albert, 1992; Ney, 2021). The dynamics of NRQM (the Schrödinger equation) is linear, deterministic, and unitary. That is, states of superpositions are preserved in their evolution. The interaction with other systems (e.g., a measurement apparatus, an observer, an observer’s friend) just makes things worse, uncontrollably extending the chain of superpositions. Tim Maudlin (1995) explains the measurement problem (the problem of ‘outcomes’ in his view) as holding three mutually inconsistent assumptions. Not solving the measurement problem is, in his view, to then endorse an inconsistent theory. Both OS and TQM claim that they do not intend to solve the measurement problem. Therefore, it is not clear the empirically successful and coherent physical theory they are being realist about.

A subsidiary problem for neutral approaches is that any solution of the measurement problem implies to endorse any of the ontic, speculative interpretations, or amending the theory somehow (e.g., introducing an ad-hoc collapse postulate).^11^ Both OS and TQM are aware of this and try to provide a non-speculative, neutral way out. I think they fail. OS relies on decoherence to avoid the speculative disagreement between a collapse/non-collapse dynamics (that is, speculative disagreement about how the measurement problem can be solved). TQM surreptitiously introduces the collapse postulate, which is a speculative solution to the measurement problem. Let me address them in tandem.

Fraser and Vickers rely on decoherence to escape speculation about whether the quantum-mechanics dynamics should feature collapses or not. Following Joshua Rosaler (2016), they say that “decoherence theory allows us to make considerable progress in recovering classical trajectories from quantum theory in an interpretation-neutral way” (Fraser & Vickers, 2022: 19). Later on, they associate decoherence with the spirit of the overlap strategy: “Thus, decoherence theory captures a substantial and important overlap in the dynamical content of all the three of our interpretations” (Ibidem). Along with it, OS suggests that different ontic interpretations enact different mechanisms to account for outcomes that can be seen as ‘effective collapses’, if it is accepted to use the term in an abstract, broadened way.

The problem is that decoherence does not work *alone* without assuming some elements of speculative, ontic interpretations. To be clear, decoherence is an important tool to understand the connection between the quantum-mechanics formalism and the macroscopic world, but only *after* having solved the measurement problem; that is, after having said *how* the quantum-mechanics formalism connects with the macroscopic world (e.g., how the problem of the outcomes can be solved). Fraser and Vickers do not say so much about how decoherence would work, but they say that.

[i]t describes how the coupling of a quantum system to a large number of environmental degrees of freedom leads the components of its wave function to separate into weakly interfering ‘branches’ that can be approximately identified with classical trajectories (Fraser & Vicker, 2022: 19)

Indeed, decoherence theory studies the correlations between a system and its environment, and how classicality “emerges” out of the quantum domain through these correlations. However, this is only possible when some speculative, ontic ingredients were previously introduced. Decoherence, per se, is a process that cannot escape the unitary and linear Schrödinger evolution and standard rules for entanglement –if a quantum system in the superposition of some observable (say, energy) interacts with an environment, the new quantum state is still in a superposition of the observable energy. Decoherence *alone* cannot account for definite outcomes because it is not its job to do so. Michael Esfeld and Antonio Vassallo (2015) emphasize this as follows:

It is the interpretative framework that sets the explanantes [sic] in which decoherences enters, and hence decoherence alone cannot be said to explain anything in a physically interesting sense (Esfeld & Vassallo, 2015: 1536)

In Fraser and Vickers’ phrasing of decoherence, they refer to the system, on the one hand, and the environment, on the other. But if a quantum system interacts with an environment, the entangled quantum state can no longer distinguish which ‘part’ corresponds to the system and which ‘part’ to the environment. Decoherence does not provide a criterion to tell them apart (see Fortin & Lombardi, 2017: 1425). Another speculative, ontic assumption is that the physical state of the system is completely given by its own individual quantum state, which is not accepted by advocates of the pilot-wave theory.

To sum up, OS is explanatorily deficient if it does not solve the measurement problem because it is unable to say how the quantum-mechanics formalism connects with the physical world. Relying on decoherence theory does not help since it can only work if a speculative, ontic interpretation was already adopted as a framework within which decoherence can work. My worry is twofold: No ontology survives; few is left to scientific realism to cling onto.

TQM in turn relies on a vague, general concept of collapse and decoherence (Egg, 2021: 16; see also fn. 17). But it is not totally clear how it addresses the issue. In some parts, Egg claims that TQM does not intend to solve the measurement problem. But in other parts, he states that the concept of ‘collapse’ is intrinsic to quantum-mechanics textbooks since their dependence on the notion of measurement (Egg, 2021: 23), although he recognizes that it is too vague a notion. Despite all the speculative divergences in more fundamental quantum theories about what a collapse *really* is, Egg departs from quantum-mechanics textbooks to note a striking convergence of how speculative proposals describe collapse on the effective level. Egg says: “My claim was that the adherents of all these approaches can agree on certain commitments concerning the reality of spin, *wave-function collapse* and wave packets” (Egg, 2021: 22. Italics mine). After analyzing the Stern-Gerlach experiment in Everett quantum mechanics and collapse theories, he says:

We have seen above that the two accounts agree in their description of what happens before the collapse. We now realize that there is a plausible sense in which they also agree about what obtains once decoherence has done its job of generating (…) different branches with a unique measurement between these two stages (Egg, 2021: 16)

I have already said the problems I found on relying on decoherence to escape speculative, ontic interpretations. It simply does not work because it needs to work within some speculative, ontic interpretation to begin with. But it is also worth noting that the collapse postulate, as it appears in quantum-mechanics textbooks, *is* already a solution to the measurement problem. It simply states that any Schrödinger-like evolution needs to be interrupted to get definite outcomes upon measurements for superposition states. It does not help much to add that the collapse is ‘effective’, or that the concept is just vague and must be accepted as it is. Suppose a Stern-Gerlach experiment oriented in the *z*-direction and a quantum state prepared in a superposition of spin in *z*. NRQM without the collapse postulate says that once the quantum system interacts with the Stern-Gerlach apparatus, the quantum state of the composite system becomes entangled. It now comprehends a quantum system in a *z*-spin superposition *and* a Stern-Gerlach apparatus in a superposition state, too. If an ad-hoc postulate is introduced the superposition is broken, and definite outcomes can be recovered. But this is precisely a solution to the measurement problem. It is a bad solution, but a solution after all. The qualification of ‘effective’ or ‘vague’ does not solve anything–effective or non-effective we are already well inside the speculative terrain.

However, the problem is even more serious. Measurement-induced collapses are full of conceptual problems that TQM cannot solve (and probably does not want to solve). This might look a bit disappointing, because a massive number of criticisms to measurement-induced collapses (see, for instance, Bell, 1990; Albert, 1990; Dickson, 2007, Lombardi et al., 2011) have been raised in the last decades, which have shed light on our understanding of quantum systems and measurements (although they didn’t boost NRQM empirical content). TQM seems to be a setback in this respect –if there is a well-grounded agreement in the field of quantum ontologies is that measurement-induced collapses are *not* a good solution to the measurement problem. To qualify them as ‘effective’ conceptually does not change its problems, but it only leaves us with a big question mark. It does not help the realist either—is it epistemically risky for the scientific realist to be ontologically committed to, say, spontaneous localizations in GRW models, but it is safer to be ontologically committed to an intendedly vague notion of ‘effective’ collapse?

The maneuver can be defended by endorsing a strong naturalistic deference to quantum-mechanics textbooks–the adoption of measurement-induced collapse just follows from the Quinean-Carnapian meta-ontological framework that TQM presumes. If wave-packet collapses appear in textbooks, then we should be realist about them and introduce them as part of our flat ontology. This way out seems desperate to me though –after all, why quantum-mechanics textbooks introduce measurement-induced collapses to account for determinate outcomes might be just a sociological fact based on pure pragmatism; it is probably easier, it is probably more effective, it is probably pedagogical convenient to get to solutions to practical problems more straightforwardly, etc. In any case, none of these answers should ever serve as guides for quantum ontology. After all, physical theories (and NRQM is not an exception) introduce lots of resources and structures that are not necessarily truth conducive. However, TQM lacks the resources “to sieve” ontological commitments (as Saatsi, 2022 points out). The way to order and clarify the ontological abundance that a Quinean ontology may bring about is by introducing structured ontologies, and thereby, speculation.

A more serious defense might run as follows. To say that collapses are ‘effective’ is what is really doing the work here, and it is asking for a more fundamental explanation that that what should be avoided. But what does this mean in an ontological proposal where everything is effective? Bohmian Mechanics also regards collapses as effective, but in the Bohmian ontology that makes sense because not everything is effective (in Bohm’s version, there is an ontologically clear sense in which collapses never occur). Alternatively, it may be argued that collapses are rather an emergent feature of NRQM. This move is similar to that of Everettian Many Worlds when relying on decoherence. This is of course a good way to interpret measurement-induced collapse and dissipate its problems, but it is not a resource available in TQM. First, it needs Everettian Many Worlds to work. Second, we need to make sense of “an emerging process” in an emergent structure. But nothing in the quantum–mechanical textbook could give us any hint to build upon the idea that wave-function collapses are somehow emergent. In quantum–mechanical textbooks, measurement-induced collapses *just* happen when quantum systems interact with measurement devices. It is an axiom of the theory. To conceive some posits or processes *within* an ontology as emergent will imply a structured ontology and further speculation.

A final point can be raised. TQM could adopt ‘effective’ collapses as part of the overlapping between different speculative accounts. In this sense, TQM is not speculating in reporting that speculative proposals involve, somehow, collapses. What remains speculative is whether collapses should be viewed as fundamental or not, but it is no longer speculative that they somehow exist. For GRW, collapses will be fundamental, but for Bohmian Mechanics or Everettian Many Worlds they will be effective.^12^ In so far as TQM *reports* what alternative speculative proposals say, this point is right—all of them, in one way or another, speak of collapses. But I am afraid that this washes out to a good extent the role of ‘effective’ collapses in TQM. To begin, all the speculative quantum theories are solutions to the measurement problem. Their solution can depend on collapses, or not. So, effective collapses appear in, say, Bohmian Mechanics as a “pragmatic affair” *because* the theory already solves the measurement problem without relying on collapses. The same goes for Everettian Many Worlds. In GRW, collapses are rather fundamental *because* they solve the measurement problem. It is true that whether collapses are effective or fundamental is a speculative question, but it is not clear to me what role collapses would play in a theory in which they do not solve the measurement problem, nor is there any other mechanism to solve the measurement problem. It is in this sense that collapses become vacuous. To put it differently, if TQM merely reports an overlapping in alternative quantum theories, what is the relevance of collapses? If they are not a solution to the measurement problem, then TQM cannot be a coherent scientific theory; if they are a solution to the measurement problem, then TQM steps into speculations since collapses cannot be just ‘effective’ (at least in the same sense as they are ‘effective’ in other quantum theories).

### Unclear ontology and triviality

My final caveat relates more closely to the assumed meta-ontology. One of the central issues of neutral approaches stems from its Quinean-Carnapian meta-ontology –it seems only to be concerned with existence questions. The problem with this is that debates about existence in quantum ontology are frequently uninteresting. And probably deeper ontological debates are uninteresting (or off-topic) for the scientific realism debate (see Saatsi, 2022: 3, 4 for comments along the same line). The latter rather center in the places and the relations of the existents within an ontology. Imagine you prepare a fermion in a superposition of spin in the *x* direction. So, you say “my fermion is in a superposition of spin in *z*” or “whereas fermions have ½-spin, bosons have integer spins”. Then, you ask: do ½ spin-fermions exist? Or does the property spin exist? Instrumentalists and Constructive Empiricists could resist the positive answer, but they are not in the business of building up a quantum ontology, which already presupposes fermions do exist.

Existence is not really the issue in quantum ontology debates, because it is almost always true that *most* of the entities, properties, states, behaviors, relations we can come up with, exist *in some sense*. What is not trivial is how ‘in some sense’ must be spelled out; *how* they exist, *where* they should be located, and *how* what exists glue together. The confusion here is to mix questions that are relevant for debates between scientific realism versus instrumentalism with those relevant for quantum ontology. Whereas neutral approaches are attractive proposals (although not new) to defend some form of selective realism against instrumentalism (e.g., against the instrumentalist charge of metaphysical underdetermination), it falls short to provide a quantum ontology. In this latter sense, it becomes almost trivial since it mainly consists of drawing up a long “old-shopping list” (to borrow Jackson’s expression) about what there is according to NRQM.

In this respect, the conservative track has been greatly superior. But they need a different meta-ontology that can provide enough resources to explain how the quantum-mechanics formalism connects with the physical world, to tell a story of what the quantum world is like. Neutral approaches cannot do it, they offer no understanding about what the quantum world is like, if NRQM is (approximately) true. A quantum ontology should at least try to do it.

## In defense of speculation –a quantum *structured* ontology

Both OS and TQM get off the conservative track. I have suggested that the conservative track, probably unwittingly, presupposes a different approach to quantum ontology. I now turn to this.

Which is the alternative meta-ontology to Quineanism and Carnapism? Any alternative that fosters the view that a substantive ontology must be structured, and that speculation plays an indispensable role there. Quineans and Carnapians fairly represent the naturalist spirit of metaphysics from the 1950s on, in common agreement against traditional metaphysics. But the naturalist spirit that has guided meta-ontology in the last decades has proved to be incapable of not only addressing basic ontological questions in physics, but also of offering a coherent picture of the natural world. Speculation in quantum ontology stems from such difficulties, not from philosophers’ lofty desires. This has not been probably explicit in the literature, but, in one way or another, most approaches to ontology in physics have sooner or later departed from the strict naturalist recipe of the Quinean-Carnapian framework, adopting a good dose of controlled speculation. The reason, to my mind, is clear –to be realists (even in its selective variety) about quantum mechanics is not enough for ontology, that is, for telling a story about what the natural world is like in the quantum regime.

Which are then the hallmarks of this alternative approach to quantum ontology? To begin, ontological questions are not primarily existence questions. Secondly, an ontology cannot be a list of beings. Frank Jackson makes the point very clearly:

Metaphysics, we said, is about what there is and what it is like. But of course, it is concerned not with any old shopping list of what there is and what it is like. Metaphysicians seek for a comprehensive account of some subject –the mind, the semantic, or, most ambitiously, everything – in terms of a *limited* number of more or less basic notions (…). (Jackson, 1994: 25. Italics mine).

The crucial word here is ‘limited’. In Jackson’s quote what gives comprehension to metaphysics is not the capacity of *listing* ontological commitments, but that of accounting for them in terms of a reduced, meager number of *basic* constituents. To do that, we need some criteria that pick such basic constituents out. This is in line with what I have called ‘intra-fundamental’, in terms of a CMB relative to a theory. That is, we need a structured ontology.

Of course, existence questions appear at some point in a structured ontology since a place for non-existents might want to be retained. But its importance must not be overstated –existence questions are just the starting point; what delivers comprehension is the structure imposed upon existents. In this sense, metaphysicians are frankly permissive with respect to what exists since existence questions are almost never directly at issue. Even though there are more and less liberal approaches to this, the idea is well represented by Armstrong’s notion of “ontological free lunch”:

Whatever supervenes or, as we can also say, is entailed or necessitated (…) is not something ontologically additional to the subvenient, or necessitating, entity or entities. What supervenes is no addition to being (Armstrong, 1997: 12)

If ontology were only about existence questions, then it would probably bring about super-abundant ontologies, and, eventually, a lack of comprehension. The way to avoid that is to strive for structured ontologies.

Let me give you two entirely divergent examples. Jonathan Schaffer’s Neo-Aristotelian view (Schaffer, 2009) takes ontology as primarily concerned about *modes* of existence. Questions like “does *x* exist?” yield the trail to questions like “*how* does *x* exist?” (Corkum 2008). Setting aside its modal commitments, it is no longer a threat to be too permissive about what exists, provided that what exists depends on (or is grounded in) a more fundamental sparse basis. Note that this directly tackles one of Saatsi’s criticisms to the TQM: the problem is not that it is overly promiscuous, the problem is that it is unable to make the necessary ontological distinctions to make promiscuity harmless. To do this, it is then necessary to change the target of ontological enquiry from existence questions to how-questions. David Lewis’ Humeanism, which dispenses with modal commitments, is also an approach to ontology that delivers a structured ontology, where the basic ontology is given by the Humean mosaic, upon which derivative content, laws and modality supervene. What the Humean mosaic is going to be like cannot be just read off from physics, since nothing there speaks of, for instance, natural properties. The problem is now to locate the derivative content in the basic content.

The point to be stressed is that, although the metaphysical content may well come from physics, the required structure comes from elsewhere. The structure requires some discrimination between what’s fundamental *within* the ontology and what is not. This cannot come from empirical sciences because it is frequently unclear in a scientific theory what depends on what, or what refers to what, and what plays a heuristic, epistemic role. So, such a task must come from philosophical exercise, which necessarily requires speculation along with some distance from empirical sciences. In other words, no physical theory *qua* empirical theory can ever fully characterize a structured ontology, because the means to do it lies far beyond what empirical sciences can deliver.^13^ By blocking speculation and by striving for neutrality, the quantum ontology cannot be substantive, but shallow and pledged of well-known problems.

Structured ontologies are nothing new in quantum ontologies: all the alternative quantum theories we have entertained for the last five decades can successfully address the main ontological issues *because* they have aimed to provide *structured* ontologies. This has not been stressed enough in the literature and many of the supporters of alternative theories may well be not even aware of this, but the most successful proposals “sieve” NRQM to separate what’s fundamental and what’s not in the intra-theoretical sense; they add additional structures to relate what’s fundamental to what’s not (if necessary); they apply various external criteria to take ontological decisions, which almost always are non-empirical. These are quantum ontologies because they strive for singling out a CMBs, not only for making safe ontological commitments. Note that nothing entails that they must engage with a sense of fundamental simpliciter, or with the idea that the alternative quantum ontologies must be the last word about what the fundamental level of reality is. But they do require some notion of intra-fundamental.

Consider Everettian Quantum Mechanics (EQM, henceforth) as introduced by Simon Saunders (1993) and David Wallace (2003, 2010, 2013). Though EQM intends to be a *literal* interpretation of the bare quantum formalism, it goes beyond that. For EQM’s defenders, quantum ontology comprehends a fundamental level, given by the universal quantum state; a multiverse level, given by the whole emergent Everett multiverse; an Everett world level, given by a single Everett world; and the level of special sciences. This highly structured ontology allows EQM not only to (dis)solve the measurement problem, but also to bridge the gaps between the quantum formalism and a layered reality. Of course, EQM oversteps what the bare formalism strictly says, but this overstepping is necessary to build a complete ontological narrative. In no place does EQM validity require us to assume that NRQM is not an effective theory. This is not the point for quantum ontology. The point is that the fundamental/derivative distinction intra-ontology is necessary to make such a narrative possible.

Consider now GRW models, which modify the quantum dynamics to account for determinate outcomes –quantum systems evolve generally according to the Schrödinger equation, but they also undergo spontaneous collapses. By this modification, GRW models foremost attempt to solving the measurement problem in a non-ad-hoc way. However, as Giancarlo Ghirardi himself recognized (Ghirardi et al. 1995), GRW models are not satisfactory if a primitive ontology is not given. The main role of the primitive ontology is not to pin a fundamental ontology down simpliciter, but to bridge the gap between the quantum formalism and the macroscopic world. For GRW models, two primitive ontologies have been proposed –a mass-density field and a flash ontology. The fundamental ontology is introduced here as a way to be clear about the beables GRW models are ultimately about, upon which the rest of the derivative ontology relies. Once again, in no place GRW needs the assumption that the theory is not effective; nor that the ontology is fundamental simpliciter. Indeed, all the attempts to make GRW compatible with relativity speak against its fundamentality simpliciter.

To sum up, most alternative quantum theories in the market single out a CMP that provides a complete description and explanation of the rest of the ontology. This explains why I take it to be a ‘conservative track’, although it has not been explicit. All of them consider NRQM as an effective theory and consider their ontologies as empirically revisable as well, but they do require their ontologies to be structured –otherwise, their purposes are futile. One could say that this is precisely the kind of speculation we should avoid; but it is this form of speculation which allows for structured ontologies. And structured ontologies are what make quantum mechanics comprehensible and in contact with the physical world. It is then speculation which delivers ontological *understanding* about the quantum–mechanical world. If we think that the target of ontological inquiry is a flat quantum ontology, then we lose ontological understanding of the quantum mechanical world; if we don’t want to lose ontological understanding of the quantum mechanical world, then we must recommend that ontological inquiry aims at a structured ontology. If this is so, then a speculative ontology seems unavoidable.

## Final remarks

I do recognize that neutral approaches do a good job in resisting anti-realist arguments based on the problem of underdetermination in quantum mechanics. Yet, I have argued that they fall short in providing us with anything close to a quantum ontology. This just emphasizes how much the problem of a quantum ontology and the problem of scientific realism in the quantum domain are orthogonal to each other. What could be a good strategy for the latter, could be a bad strategy for the former. I think that neutral approaches are bad strategies for quantum ontology because the sort of meta-ontology that they ultimately put forward—the Quinean-Carnapian framework yields at best flat, structureless ontologies. Such ontologies, I have claimed, are not only insufficient to solve the main ontological issues in philosophy of quantum mechanics but are not enough for a quantum ontology. A substantive quantum ontology, if in the business of delivering understanding, does not require to talk about what is fundamental simpliciter, but it needs for a structure, for a distinction between what is fundamental and what is not *within* the ontology. That is the target of ontological inquiry and that is what the conservative track has been doing so far. And it is what, I believe, should continue to be done in the future.

## Data Availability

Not applicable.

## Footnotes

1 I use the name ‘non-relativistic quantum mechanics’ as referring to theory formulated in the Hilbert-space formalism, where quantum states are represented by density operators ($\rho$), pure states as vectors on the Hilbert space ($\left|\psi\right.\rangle$), and observables as Hermitian operators. Probabilities are extracted from the theory through the Born Rule and the evolution of quantum systems is generally unitary, deterministic, and linear according to the Schrödinger equation. However, NRQM when introduced in textbooks usually involves the Collapse Postulate, which prescribes that (pure) quantum states undergo a stochastic collapse when measured. To avoid confusions, I reserve the name ‘NRQM’ for a formulation of the theory without the collapse postulate. The introduction of the collapse postulate, as in the text-book approach, is already a solution of the measurement problem (see fn. 3).

2 The name is taken from the Symposium “Quantum Realism: moving to neutral” at the *Biennial Meeting of the European Association for Philosophy of Science* (Turin, 2021).

3 The measurement problem is frequently understood in two different ways. On the one hand, it is a problem about ‘outcomes’ (Albert 1992; Maudlin 1995) –how to account for *determinate* outcomes when non-relativistic quantum mechanics predicts, in many cases, that one should observe *indeterminate* outcomes. On the other, it is a problem about ‘measurement-induced collapses’. I think that the former formulation is more general and must be preferred.

4 An analogous distinction could be drawn in terms of *absolute* and *relative* fundamentality. In my vocabulary, intra-fundamentality will be a type of relative fundamentality, but it emphasizes the intra-distinction necessary to build a structured ontology. I thank an anonymous reviewer for suggesting this clarification.

5 In general philosophy of science, the problem of underdetermination of theories by empirical data (*contrastative underdetermination)* is a very well-known threat for scientific realism. In brief, the idea is that a set of empirical data can be accounted for alternative, different scientific theories (see Duhem 1974, van Fraassen 1980, Cushing 1994). Then, if scientific realism submits that we ought to take our scientific theories as (approximately) true, genuine underdetermination entails that the same empirical data is compatible with, at least, two rival theories, which might potentially involve unalike views of what the world is like. This is a problem of theory choice, for which not not only logical consistency and empirical adequacy are necessary, but also various supra-empirical virtues (Laudan 1990; Laudan and Leplin 1991).

6 Callender (2020) referred to Cordero’s objectivist quantum mechanics as TQM. Even though some general features can be already found in his proposal, it is not quite clear that Cordero truly defends a textbook-based approach to quantum ontology. To my mind, he does offer general criteria for selective realism, without stepping into ontological debates.

7 *Effective theories* are considered to be valid only at a given “level” or “regime” (e.g., the low energy regime vs. high energy regime). Thus, we are presented with a “tower of theories”, each of these framed in terms of appropriate parameters for a particular level, describing the relevant interactions at that level, and that breaks down when we jump into a different level.

8 For Quineans, some sort of structure can be found in the *ideology* (what ideas are expressible in a theory), not in the ontology.

9 It might be asked if further (speculative) criteria should not be added to form a neutral basis of agreement. For instance, the existence of the electric potential in classical electromagnetism is contested on the basis of symmetry arguments. However, additional reasons must be provided to show why ontological commitments must follow symmetry arguments, which could lead us to incur in speculation. I thank an anonymous reviewer for pointing to this case as an example.

10 It is worth noticing that other quantum-mechanics textbooks offer alternative relations between operators, observables, and space–time symmetries.

11 It is worth mentioning that there are epistemic and pragmatist solutions to the measurement problems that do not incur in ontic, speculative proposals (see, for instance, information-theoretic interpretations, Quantum Bayesianism, and QBism). But, as they do not attempt to provide a quantum ontology, I have not included them in this discussion.

12 I thank an anonymous reviewer for raising this point.

13 Eugene Wigner says: “it is clear that, in this sense, physics does not endeavor to explain nature. In fact, the great success of physics is due to its restriction of its objectives: it only endeavors to explain the regularities in the behavior of objects” (Wigner 1964: 6).
